# Push and pull factors influencing preference for traditional healing by jaundiced patients in Bangladesh

**DOI:** 10.1371/journal.pone.0312962

**Published:** 2024-11-01

**Authors:** Md. Ismail Gazi, Hema Binte Hamid, A. B. M. Alauddin Chowdhury

**Affiliations:** 1 Department of Public Health, Daffodil International University, Dhaka, Bangladesh; 2 Department of Gastroenterology, Sheikh Russel National Gastroliver Institute and Hospital, Dhaka, Bangladesh; 3 Kamarkhanda Upazila Health Complex, Sirajganj, Bangladesh; BOKU: Universitat fur Bodenkultur Wien, AUSTRIA

## Abstract

**Background:**

Jaundice is a significant health issue in Bangladesh. Many patients choose traditional medicine (TM) over conventional medicine (CM) for treating jaundice. This study aimed to identify and analyze the push and pull factors influencing the preferences of jaundiced patients for traditional healing methods in Bangladesh.

**Methods:**

This cross-sectional study used a mixed-methods approach. Two focus group discussions (FGDs) were conducted with 16 participants (8 per group) who had experience of using traditional medicine for jaundice. In-depth interviews with five traditional healers were carried out to gather qualitative insights from the healers’ perspectives. Quantitative data were collected from 400 jaundiced patients at a tertiary-level hospital using a semi-structured questionnaire. Chi-square tests and binary logistic regression were used to analyze associations between sociodemographic factors, push-pull factors, and treatment preferences.

**Results:**

The study found that 62% of participants favored TM for jaundice treatment. The scalp and hand cleansing rituals (46%), herbal remedies (37%), garlanding (23%), and use of talismans or amulets (21%) were the most common practices. Qualitative analysis revealed five push factors, eight pull factors, four intervening obstacles, and five personal factors that influenced jaundiced patients’ preference for TM. Patients also emphasized the significance of spiritual and emotional aspects in their decision-making process when choosing TM. Significant associations were observed between treatment preference and age group (p = 0.002), residence (p = 0.018), education level (p<0.001), and religion (p = 0.015). Individuals aged 50 years and above (70.9%), rural residents (68%), and those with no or primary education (72%) were more likely to opt for TM. Key push factors towards TM included high costs of CM (OR: 6.80, 95% CI: 2.10–22.04) and perceived ineffectiveness of CM. Strong pull factors were accessibility of TM (OR: 11.18, 95% CI: 4.03–31.00), perceived effectiveness of TM (OR: 3.45, 95% CI: 1.05–11.37), personal testimonials (OR: 7.55, 95% CI: 2.75–20.69), lower costs of TM (OR: 10.48, 95% CI: 4.30–25.54) and lack of information about conventional treatments for jaundice (OR: 13.82, 95% CI: 4.62–41.33).

**Conclusion:**

The study reveals that both push and pull factors influence jaundiced patients in Bangladesh to choose TM over CM, with decisions shaped by personal, socioeconomic, and geographical factors.

## Introduction

Jaundice is a medical condition characterized by yellowing of the skin and sclera (white portion of the eyes) due to elevated bilirubin levels in the blood, typically becoming noticeable when serum bilirubin exceeds 3 mg/dl [1]. It is classified into three types: pre-hepatic, hepatocellular, and post-hepatic [1]. In Bangladesh, jaundice is widely referred to as *Jaundish*, and is locally classified into various types depending on the perceived underlying causes or symptoms. These include *liver jaundish* (hepatic jaundice), *rokto jaundish* (blood jaundice), and *mete jaundish* (characterized by a pale discoloration of the body).

While common in newborns, jaundice in adults often signals serious underlying conditions such as hepatitis, liver cirrhosis, or pancreatic cancer [1]. In resource-limited countries like Bangladesh, where fecal contamination of drinking water is common, Hepatitis E (HEV), Hepatitis A (HAV), and acute Hepatitis B are the most common causes of acute jaundice [2, 3]. In cases of obstructive jaundice in adults, frequent causes include choledocholithiasis, carcinoma of the hepatobiliary system or pancreas, bile duct strictures, retained stones, and parasitic obstructions [4]. Jaundice patients are at risk of liver dysfunction, renal failure, cardiovascular issues, malnutrition, bleeding tendencies, compromised immunity, wound complications, and increased morbidity and mortality [5]. Although Hepatitis A and E are typically self-limiting, Hepatitis E poses significant risks during pregnancy, potentially leading to postpartum hemorrhage, miscarriage, perinatal mortality, and encephalopathy [6, 7].

Traditional medicine (TM) is widely used globally for treating various diseases. Many studies have highlighted the beneficial use of TM in addressing conditions such as diabetes, liver diseases, sexual dysfunction, infertility, malaria, and cancers, particularly prostate, cervical, breast, and skin cancers [8–12]. TM is also deeply rooted in Bangladesh’s cultural, religious, and historical practices and is broadly recognized as a component of complementary and alternative medicine (CAM) in the country. Studies found that one-third of the population uses CAM for the management of chronic illnesses [13, 14]. The widespread utilization of traditional healthcare is rooted in its effectiveness, accessibility, affordability and less adverse effects [14]. For treating illnesses like jaundice, TM is perceived by the general population as an effective and less harmful alternative, leading many to prefer it over conventional medicine (CM) [7]. These traditional methods typically involve herbal remedies using various plant parts, as well as spiritual and supernatural practices for diagnosis and treatment [15]. Such methods are widely accepted throughout the country, especially in rural areas and among people with low socioeconomic status [15]. Although modern medical science has advanced, the persistent preference for traditional jaundice treatments in Bangladesh raises critical questions about the underlying factors driving such choices. Therefore, this research aims to identify and analyze the push and pull factors influencing jaundice patients’ preferences for traditional healing methods over conventional medical treatments.

While numerous studies have examined traditional medicine (TM) in Bangladesh and neighboring countries, research specifically focused on jaundice and the healthcare-seeking behaviors associated with its treatment remains limited. Therefore, this study contributes significantly to public health by filling a critical gap in understanding the healthcare-seeking behaviors of jaundiced patients and advancing the existing research on medical pluralism in this region. Given the high prevalence of jaundice and its potential complications, especially in resource-limited settings, understanding the push and pull factors influencing treatment choices is crucial for designing effective health interventions and health education campaigns. Moreover, the insights gained from this study can help healthcare providers tailor culturally sensitive approaches, which are crucial in improving patient engagement, reducing reliance on some potentially harmful traditional practices and promoting informed decision-making for more appropriate treatment options. Moreover, it contributes to medical anthropology by exploring how cultural factors influence health practices in Bangladesh, which could also help shape global health strategies for other countries with similar socioeconomic profiles.

### Theoretical framework

The foundation of our study was based on Everett Lee’s push-pull theory of migration [16]. Everett Lee developed a comprehensive migration theory in 1966, outlining the elements that drive migratory movements and categorizing them as push or pull factors. Push factors are unfavourable elements in one’s present location (place of origin) that urge individuals to leave, whereas pull factors are favourable elements of another area (destination) that attract people. Additionally, migrants encounter difficulties during migration due to intervening obstacles (such as political, legal, and geographical). Personal factors (such as religion, education, income level, health, age, perception, etc.) are subject to significant variation among individuals and can either promote or deter migration, depending on each individual’s specific circumstances and perceptions.

This study adapted Lee’s model to identify and analyze the push and pull factors influencing TM use. The study also examined possible barriers that people could encounter while seeking TM. Using Lee’s migration theory in the context of TM, this study detailed the determinants influencing healthcare decisions among various populations in Bangladesh.

## Methods

This cross-sectional study was conducted from December 2023 to April 2024 using a mixed-method design to assess the factors influencing the choice of traditional treatments for jaundice. The key informants for the study included adult community members and jaundiced patients with prior experience using traditional medicine, as well as traditional healers. Qualitative data was collected from 16 participants (8 in each group) through two focus group discussions (FGDs) and interviews with five traditional healers. A 1:1 male-to-female ratio (8 male, 8 female) was achieved in FGDs through purposive sampling, ensuring a balanced representation of genders. Five traditional healers were recruited through snowball sampling. Quantitative data was collected from jaundiced patients (clinically confirmed by physicians) who were aged 18 years or older, of either sex and represented a balance of residence and gender. Data collection took place at the Sheikh Russel National Gastroliver Institute and Hospital, a tertiary-level facility specializing in gastrointestinal and hepatobiliary diseases that serves patients from across the country.

### Operational definitions

#### Traditional medicine

The World Health Organization define TM as "the sum total of knowledge, skills and practices based on the theories, beliefs and experiences indigenous to different cultures that are used to maintain health, as well as to prevent, diagnose, improve or treat physical and mental illnesses" [17]. In this study, traditional medicine and traditional healing have been used interchangeably.

#### Conventional medicine

The National Cancer Institute (NCI) defined CM as "a system in which medical doctors and other health care professionals (such as nurses, pharmacists, and therapists) treat symptoms and diseases using drugs, radiation, or surgery. Also called allopathic medicine, biomedicine, mainstream medicine, orthodox medicine, and Western medicine" [18].

#### Push factors

Factors that drive people away from conventional medical treatments.

#### Pull factors

Factors that attract individuals towards traditional healing methods.

#### Intervening obstacles

Factors that prevent or discourage the use of traditional methods.

#### Personal factors

Individual characteristics that influence the choice of treatment.

### Sample size

For qualitative data collection (FGD and in-depth interviews), the sample size was determined purposively to ensure the selection of participants who could provide the most relevant and rich information regarding traditional jaundice treatments. For the quantitative survey, the sample size was calculated using N = [Z^2^. p. (1-p)]/E^2^ formula derived from the standard error for proportions, commonly applied in statistics to estimate sample sizes in large populations [19]. Considering the margin of error (E) = 5%, proportion (p) = 59% [20], and confidence level (Z) = 95% (Z-score of 1.96 for a 95% confidence interval), a sample size of 372 was calculated. Final data was collected from 400 patients, slightly exceeding the calculated sample size to account for incomplete data or any unexpected attrition, ensure feasible distribution across the four strata (100 per stratum), and facilitate easier statistical presentation without introducing bias or compromising study’s feasibility and ethics.

### Data collection, validity, and reliability

**Fig 1:**
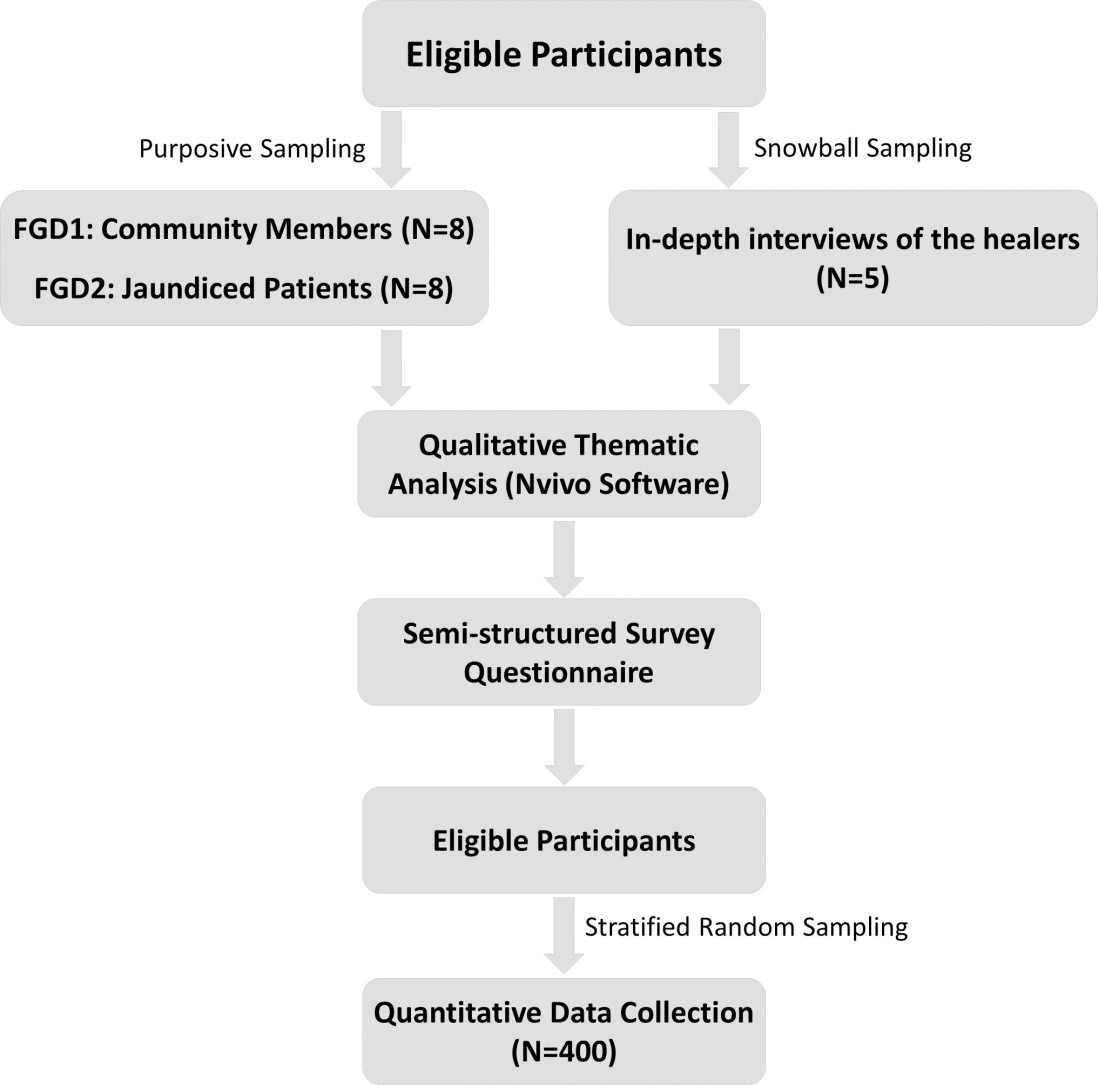
Data collection flow chart.

Data collection for this study involved two FGDs recruited through purposive sampling, in-depth interviews with traditional healers recruited through snowball sampling, followed by a structured survey of jaundiced patients using a stratified random sampling design (Fig 1). FGDs provided insight into patient perspectives, while interviews with healers offered a service provider’s viewpoint.

The first FGD included eight community members, while the second involved eight jaundiced patients who had previously sought treatment from traditional healers. Each FGD was held in a quiet, private setting to encourage open discussion. The primary objectives of these discussions were to understand (i) the factors that push individuals to choose traditional methods over conventional ones for jaundice, (ii) the factors that pull them towards traditional healing methods, (iii) the obstacles they face in accessing traditional treatments, and (iv) the personal and socioeconomic factors influencing their choice between TM and CM. The discussions were guided by a semi-structured interview guide, audio-recorded with consent, and later transcribed verbatim manually. Supplementary notes were taken to capture non-verbal cues missed by audio recordings. The transcripts were translated into English and verified by an external reviewer.

Moreover, five in-depth interviews were conducted with traditional healers selected through snowball sampling—a common technique in qualitative research for recruiting hard-to-reach populations [21]. A focus group participant helped identify the first healer who consented to participate, and each subsequent healer was recommended by the previous one. Each interview was aimed at uncovering the obstacles healers encounter in their practice.

The qualitative data were analyzed using thematic analysis with Nvivo (version 12) software to identify and categorize themes. This analysis resulted in four main themes: push factors, pull factors, obstacles, and personal factors, with 22 codes for these themes. A semi-structured survey questionnaire was developed based on the findings. Three methods were used to ensure validity: peer debriefing, participant validation, and triangulation, all of which are widely recognized for their effectiveness in qualitative research [22]. Peer debriefing was conducted with three public health and social science experts to critically examine the analytical process and interpretations. Participant validation was achieved by sharing the preliminary findings with the FGD participants to ensure their responses were accurately represented. Methodological triangulation was conducted by cross-verifying the findings through informal interviews with 30 participants selected via simple random sampling from the community. This process revealed no significant new information, confirming the consistency of the initial findings. To evaluate the reliability and internal consistency of the survey questionnaire, responses from 30 individuals were analyzed using Cronbach’s alpha, with a threshold of 0.70 for acceptable reliability. Most factors showed acceptable internal consistency, with alpha values ranging from 0.71 to 0.83. However, the holistic approach factor (0.61) and fear of side effects of CM factor (0.58) did not meet the threshold. According to George and Mallery, a reliability coefficient below 0.5 is considered unacceptable, and since none of these subscales fell below this level, they were retained in the instrument [23].

The study used a stratified random sampling design for quantitative data to ensure equal representation across four strata: Urban Males, Urban Females, Rural Males, and Rural Females, with 100 participants targeted per stratum. Medical doctors (who also served as data collectors) assessed patient eligibility using the inclusion criteria before enrollment. Once eligible, patients were stratified by gender and residence, and randomization was conducted using an Excel workbook. A random number was generated for each patient, with those below 0.5 selected for the study. The generated random numbers were also copied and pasted as values into another Excel workbook to maintain a static dataset for all selected participants. Among the selected participants, those who consented were enrolled in the study, and data collection began immediately. For effective data collection and variation, 12 participants were randomly selected each working day, with continuous monitoring of each stratum’s enrollment status. Once a stratum’s quota (e.g., 100 Urban Males) was met, no further patients were enrolled from that group. Of the 632 patients approached, 400 were included in the study. The semi-structured questionnaire collected detailed information on demographics, health history, initial actions taken, types of treatment received (traditional or conventional), reasons for choosing traditional healing, and perceived barriers to accessing modern healthcare. It also gathered data on the types of traditional healing practices sought by patients and associated costs.

### Data analysis

Qualitative data was analyzed using Nvivo software (version 12) for thematic analysis to identify the push and pull factors influencing the use of traditional healing methods for treating jaundice. For the quantitative analysis, SPSS software (version 26) was used. Frequencies and percentages were calculated to provide an overview of the participants’ characteristics. The chi-square test was used to examine the significant associations between sociodemographic factors and treatment preferences. Binary logistic regression was conducted to evaluate the influence of various predictors on the likelihood of using traditional healing methods. Both unadjusted and adjusted odds ratios (OR) were calculated to calculate the predictors’ effects, with adjustments accounting for potential confounders. A p-value of less than 0.05 was considered statistically significant.

### Ethical issue

All ethical principles were followed per the Helsinki Declaration (1964) for medical research involving human subjects and research. The FAHS Research Ethics Committee (REC) of Daffodil International University reviewed and approved the ethical clearance (Ref. no. FAHSREC/DIU/2023/SMIG-06) before conducting the study. After the study objectives and methods were explained, informed written consent for the data collection was obtained from all the participants. For participants who could not read and write, informed verbal consent was obtained in the presence of at least one witness and documented in written notes at the time of the interview per the WMA Declaration of Helsinki. The REC approved the use of informed verbal consent due to the low-risk nature of the study and the contextual consideration of the literacy rates in Bangladesh. Their participation was voluntary, and they could withdraw from the study any time. Participants were assured of their anonymity. Data was stored in a secure, password-protected database to ensure its integrity and confidentiality.

## Results

**Fig 2:**
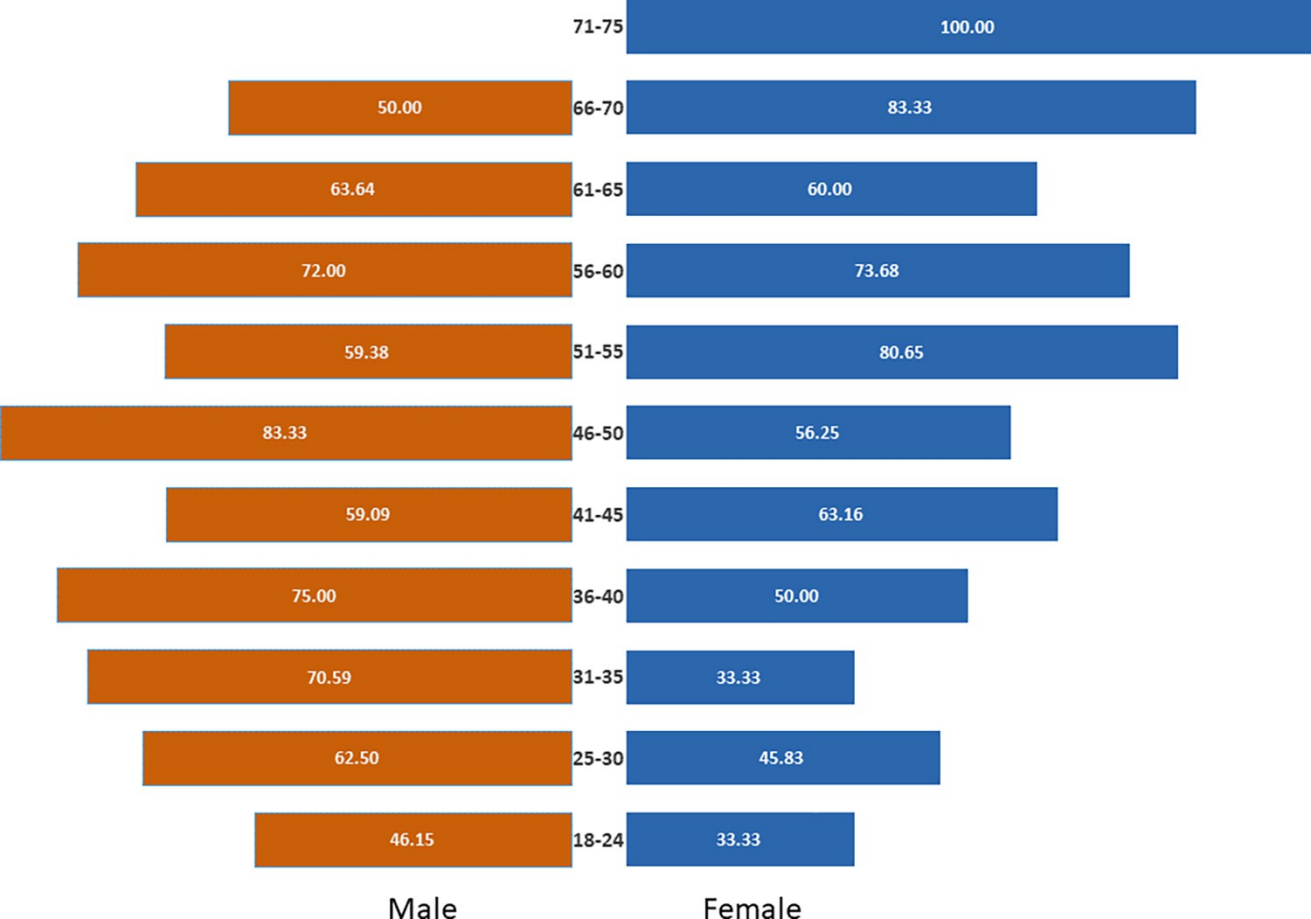
Population pyramid illustrating age-group preferences for traditional healing.

The study population comprised 200 males and 200 females, with 62% favoring TM. Older adults (71% for 50+ years), rural residents (68%), and individuals with primary education (72%) showed a higher inclination towards TM. Chi-square tests revealed significant associations between age group (p = 0.002, Cramer’s V = 0.18), residence (p = 0.018, Cramer’s V = 0.11), education level (p = <0.001, Cramer’s V = 0.25), and religion (p = 0.015, Cramer’s V = 0.12) with treatment preference (Table 1). The preference for TM increases across different age groups, as shown in the population pyramid (Fig 2).

**Table 1:** Sociodemographic characteristics of the participants and the association of the variables with TM and CM.

		Total	TM	CM	Chi-Square (TM vs CM)	P value	Cramer V
		N (%)	N (%)	N (%)			
Total		400 (100)	249 (62)	151 (38)	-	-	-
Sex							
	Male	200 (50)	132 (66)	68 (34)	2.39	0.122	0.08
	Female	200 (50)	117 (59)	83 (42)			
Age group							
	18–30	89 (22)	44 (49)	45 (51)	12.54	**0.002**	0.18
	30–50	132 (33)	78 (59)	54 (41)			
	50 and above	179 (45)	127 (71)	52 (29)			
Residence							
	Rural	200 (50)	136 (68)	64 (32)	5.63	**0.018**	0.12
	Urban	200 (50)	113 (57)	87 (44)			
Monthly Income							
	<10000	74 (19)	44 (60)	30 (41)	5.09	0.165	0.11
	10000 to 20000	165 (41)	111 (67)	54 (33)			
	20000 to 40000	118 (30)	65 (55)	53 (45)			
	>40000	43 (11)	29 (67)	14 (33)			
Education							
	No Education	136 (34)	84 (62)	52 (38)	25.16	**<0.001**	0.25
	Primary	129 (32)	93 (72)	36 (28)			
	Secondary	72 (18)	49 (68)	23 (32)			
	Higher Secondary	33 (8)	14 (42)	19 (58)			
	University/College	30 (8)	9 (30)	21 (70)			
Religion							
	Muslim	369 (92)	236 (64)	133 (36)	5.90	**0.015**	0.12
	Hindu	31 (8)	13 (42)	18 (58)			

### Influencing factors for seeking TM: Patients’ perspective

The thematic analysis of the FGD and interviews uncovered several recurring themes illuminating the complex dynamics surrounding traditional healing practices and the factors influencing decision-making when choosing treatment.

#### Perceived effectiveness

A significant theme that emerged was the perceived effectiveness of traditional treatments for jaundice. The positive outcomes experienced by relatives and community members generally encouraged others to pursue traditional methods.

Participant H2 (male, 27): *"Many people in my community have recovered from jaundice using traditional methods. It works better than medical treatments for jaundice."*

#### Personal testimonials

Testimonials from friends and family who had successful experiences with traditional healing played a crucial role in shaping patients’ decisions.

Participant C1 (Female, 25): *"My uncle was cured of jaundice after visiting a traditional healer. The healer treated him by washing his hands and head. His jaundice disappeared after 13 days. His positive experience convinced me to try it, too."*

#### Perceived safety

Some participants believed that traditional healing methods are inherently safe due to their natural origins and minimal side effects.

Participant H1 (male, 45): *"Trees, plants, herbs—these are natural. Anything natural is safe and has no side effects. Sometimes, the healer just washes the hands or puts a talisman around the neck. If I wear amulets or drink herbal juice for jaundice, I cannot see any harm in that."*

Participant B1 (Female, 30): *"If I wear a jaundice garland and it cures my jaundice*, *that’s great*. *If not*, *at least it doesn’t harm*.*"*

#### Low cost

The affordability of traditional healing makes it an attractive option for many jaundiced patients. TM is considered an affordable alternative for those who cannot afford the high cost of modern medicine.

Participant D2 (male, 50): *"Going to doctors means lots of diagnostic tests, which are costly. But, if I visit the Kabiraj (the traditional healer), he can alleviate the jaundice with very little money."*

#### Easily accessible

The proximity and availability of traditional healers, who often reside within the community, significantly contribute to their popularity.

Participant A2 (female, 30): *"The hospital is far from my house and often crowded. But the healer lives in my village, and I can see him anytime. It’s very convenient for me compared to the hospital."*

#### Importance of cultural beliefs

Cultural beliefs profoundly influence the healthcare decisions of jaundiced patients. Some spoke passionately about their heritage and traditions, viewing traditional practices as integral to their identity and ancestral connection.

Participant A1 (male, 57): *"Our fathers and grandfathers relied on this tradition, and so do we. When I had jaundice, I followed the rituals and remedies passed down through generations."*

#### Trust in traditional healers

Trust in traditional healers emerged as another central theme in the qualitative analysis. Many jaundiced patients expressed unwavering faith in the expertise and wisdom of traditional healers.

Participant E1 (female, 40): *"I’ve known our local traditional healer since I was a child. He’s like our guardian, so I have trust in him. When I got jaundiced, I knew he would take care of me. He understands our illnesses better than anyone else."*

#### Hesitancy toward modern medicine

A prevailing theme was the hesitancy toward modern medicine, with some patients expressing concerns about the impersonal nature of modern healthcare and fear of medical interventions.

Participant C2 (male, 35): *"I’ve heard many negative stories about hospitals and doctors. They don’t try to understand our needs, and they rush through everything. I didn’t want to go to the hospital for my mete jaundice because I thought they wouldn’t understand me."*

Participant A1 (male, 57): *"When my niece developed jaundice*, *she was pregnant*. *We initially consulted a doctor who suggested some tests*. *After getting the test results*, *the doctor did not prescribe any specific treatment for the jaundice; he only advised her to rest*. *But we had doubts*. *So*, *we sought a second opinion from a kabiraj (traditional healer)*. *He suggested drinking three chanted coconuts for three days*. *Her jaundice improved within three weeks of drinking the chanted coconut water*.*"*

#### Fear of stigmatization for seeking medical care too soon

The fear of being stigmatized for seeking medical care too soon was a poignant theme. Some jaundiced patients reported feeling judged or ridiculed by their communities if they sought medical treatment immediately after noticing jaundice symptoms. This social stigma acted as a powerful deterrent to seeking early medical intervention.

Participant F2 (male, 26): *"I knew I had jaundice, but I didn’t want people to talk. In our community, they say, ’Why run to the hospital for little things like jaundice? Go to Ojha, and he will emit your jaundice.’ So, I visited Ojha at first for my jaundice."*

#### Balancing cultural beliefs and modern medicine

Interestingly, a subgroup of patients expressed a desire to balance cultural beliefs with modern medicine, recognizing the value of integrating traditional and medical treatments.

Participant B2 (female, 50): *"I have respect for our traditional healing system, but I also believe in what doctors can do. I initially went to the healer but later sought medical advice when I didn’t improve. I think that the combined treatment is the best option for jaundice."*

#### Holistic approach

The holistic nature of traditional healing, addressing spiritual, emotional, and physical health, is particularly appealing to many jaundice patients.

Participant D1 (male, 57): *"Traditional healers not only treat the disease but also help me feel better spiritually and emotionally. When Huzoor (religious person) treat me by blowing over me after reciting a Dua (Islamic sacred recitation), It feels like a more complete approach."*

### Obstacles for traditional healing practices: Healers perspective

#### Legality issues

The legal recognition of traditional healing practices is a significant barrier for the healers. Most practices are not officially recognized, limiting their public acceptance and integration into mainstream healthcare.

Traditional Healer D (male, 54): *"We face significant challenges because many of our practices aren’t legally recognized. This makes it hard for us to openly provide our services and also makes it difficult for potential clients to trust our treatments."*

#### Availability constraints

Access to essential resources, including herbs and experienced practitioners, is becoming increasingly scarce, which complicates the delivery of traditional healing.

Traditional Healer B (male, 45): *"It’s becoming increasingly difficult to find certain plants and herbs that are essential for our treatments. Also, there are fewer experienced practitioners available now, which makes it harder for many people to find traditional healing."*

#### Social stigma

Disapproval from certain community members and modern healthcare providers often poses a formidable obstacle to traditional healing.

Traditional Healer E (male 60): *"There’s a lot of judgment from parts of the community and even some modern healthcare providers. This judgment makes some patients hesitant to visit us. Again, sometimes it leads to misunderstanding within our own neighborhoods"*

#### Skepticism due to lack of scientific evidence

The absence of scientific documentation supporting the efficacy of traditional methods often leads to skepticism among potential new patients.

Traditional Healer C (male 40): *"I have to admit that our healing practices don’t have scientific evidence. Sometimes, I can’t explain to my patients how my methods work to treat jaundice. I just tell them to believe in the system, and this belief will help heal the jaundice."*

### Push-pull factors for seeking traditional medicine for jaundice

**Fig 3:**
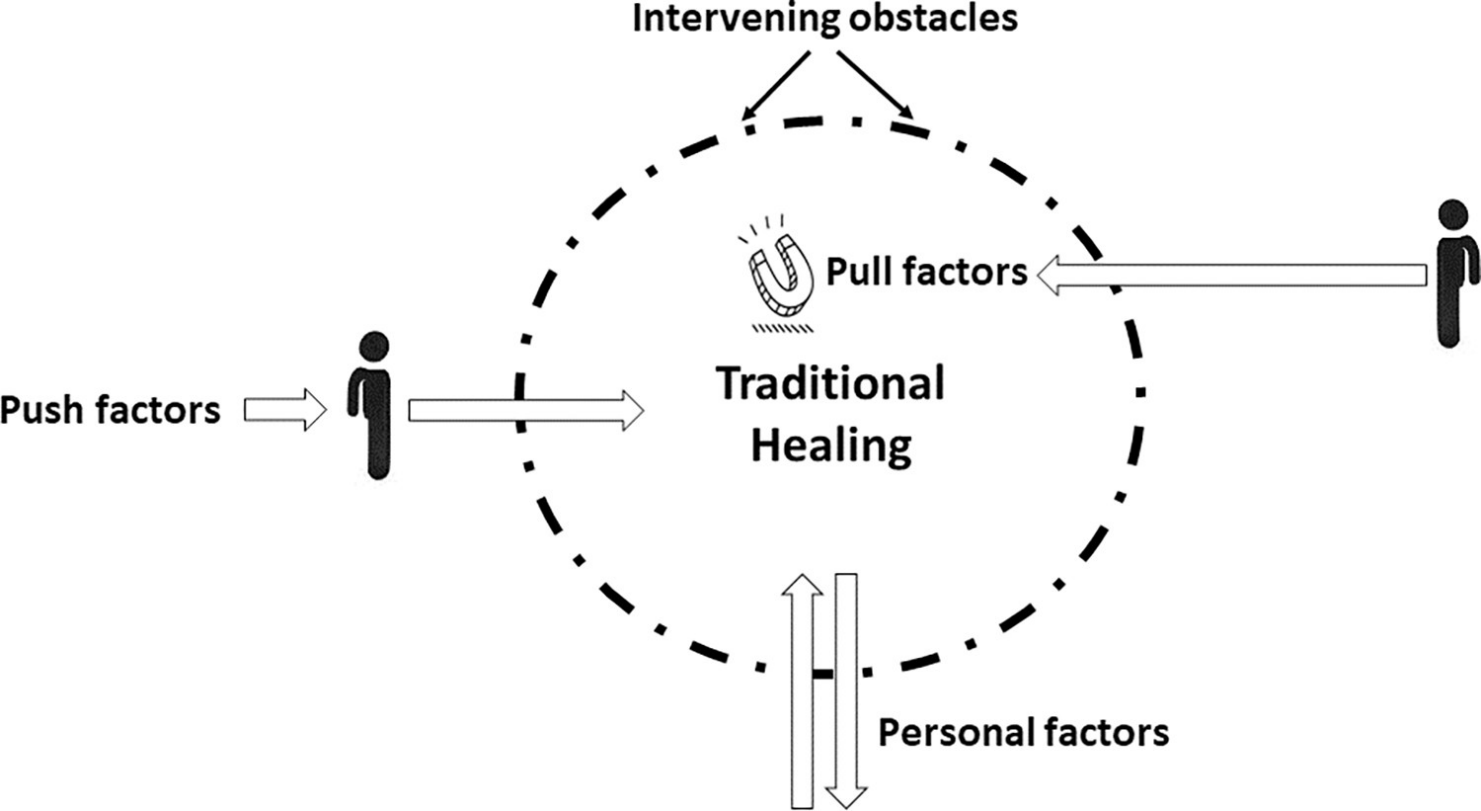
Dynamics of push and pull factors in seeking traditional medicine.

Both push and pull factors, identified from thematic analysis and participant discussions, were used to explain why patients in Bangladesh pursued traditional healing methods for jaundice (Table 2). Push factors drove patients away from conventional medical treatments, while pull factors attracted them to traditional healing. This dual perspective showed the enduring popularity of traditional healers in Bangladesh. To successfully reach out to TM, one must cross the intervening obstacles to reach the TM, while personal factors might push away or towards the traditional healing system (Fig 3).

**Table 2:** Push and pull factors influencing the pursuit of traditional healing methods.

Push Factors	Pull Factors	Intervening Obstacles	Personal Factors
Cost: Expensive conventional treatment.	Affordability: Lower cost of TM compared to CM.	Legitimacy: Most traditional practices are illegal per medical law.	Education level: Lower education influences preferences for TM.
Accessibility: Lack of nearby healthcare facilities.	Convenient accessibility: Users can readily reach the healer.	Availability: Certain herbs, practices, or practitioners are difficult to find.	Age: Older people prefer TM for jaundice more.
Perceived ineffectiveness: Belief that conventional medicine is not effective for jaundice.	Perceived efficacy of TM: TM for jaundice is believed to be more effective.	Lack of documented efficacy: Skepticism due to a lack of scientific studies supporting traditional methods.	Health beliefs and knowledge: Personal beliefs about health and wellness influence treatment choices.
Side effects: Fear of side effects from conventional medications.	Perceived safety: Belief in TM is inherently safer than CM.	Stigma in society: Condemnation from some community members or healthcare practitioners.	Previous experiences: Personal or familial past experiences with either CM or TM.
Cultural barriers: Conventional medicine does not align with the patient’s cultural or personal beliefs.	Testimonials: Positive stories from friends, family, or community members using TM.		Lack of information: Uninformed about the availability of modern treatments for jaundice.
	Cultural acceptance: TM represents local culture or family traditions.		
	Holistic approach: Preference for therapies targeting both spiritual and emotional wellness and physical ailments.		
	Religious affiliation: TM related to religious beliefs (e.g. dua, mantra) appeals to individuals who value spiritually based treatment.		

#### Prevalence of traditional healing practices for jaundices

The majority of the 249 individuals who used traditional treatments relied on multiple traditional healing methods. The most common method was the scalp and hands cleansing ritual (46%), herbal remedies (37%), jaundice garland (23%) and the use of talismans or amulets (21%). The average cost of treatment was 143 Bangladeshi Taka (BDT), equivalent to 1.21 US Dollars (USD) per exchange rate as of 30 June 2024 (Table 3).

**Table 3:** Prevalence of traditional healing methods for jaundice with average cost.

		N (%)*	Average cost in BDT (min-max)	Average cost in USD
Total TM used		249	143 (0–450)	1.21
Types of healing				
	Scalp and hands cleansing ritual	114 (46)	96 (30–200)	0.81
	Use of talisman or amulets	53 (21)	85 (30–250)	0.72
	Use of jaundice garland	57 (23)	30 (0–100)	0.25
	Herbal remedies	91 (37)	143 (50–350)	1.21
	Chanted coconut water	45 (18)	347 (120–450)	2.94
	Liver khilano	34 (14)	162 (100–300)	1.37
	Cauterization (heated metal on the forehead)	19 (8)	127 (90–250)	1.08
	Chanted banana	29 (12)	42 (30–60)	0.36
	Liver katano or Keura	10 (4)	140 (90–300)	1.19
	Chanted lemons over the earthen oven	9 (4)	108 (60–150)	0.91
	Chonga method (ear candling)	7 (3)	164 (100–300)	1.39
	Pet tana (abdominal suction with a horn)	5 (2)	142 (60–300)	1.2
	Chanted mud shower	3 (1)	267 (200–400)	2.26

*The total percentage exceeds 100% as the respondents opted for multiple treatment options

Table 4 presents the association of various push-pull factors with the use of TM for jaundice treatment, analyzed through chi-square tests, crude odds ratios (OR), and adjusted odds ratios (AOR). It reveals a complex interplay of demographic, economic, social, and informational factors. Notably, older age (50 and above) and urban residence were significantly associated with increased TM use. However, gender was not statistically significant. Economic considerations played a significant role, with the high cost of conventional medicine and the low cost of TM both strongly predicting TM use. Accessibility (easy access to traditional healers) also significantly increased the likelihood of choosing TM. Social influence (testimonials from others) strongly influenced treatment choices. Interestingly, the lack of information about conventional treatments for jaundice was the most powerful predictor of TM use, highlighting a critical gap in health communication. The perceived effectiveness of TM also played a role in treatment choice. While cultural beliefs showed some influence, their impact was less pronounced in the adjusted analysis. Religious beliefs, surprisingly, were negatively associated with TM use.

**Table 4:** Association of the push-pull factors with the use of TM and binary logistic regression.

		TM Used	TM not used	Total	Chi-square (p-value)	Crude OR (95% CI)	Adjusted OR (95% CI)
Gender	Male	132	68	200	2.39 (p = 0.122)	Ref	Ref
	Female	117	83	200		0.73 (0.48–1.09)	0.74 (0.36–1.48)
Age Group	18–50	122	99	221	10.44 (p = 0.001)	Ref	Ref
	≥50	127	52	179		1.98 (1.31–3.01)	1.85 (0.88–3.89)
Residence	Rural	136	64	200	5.63 (p = 0.018)	Ref	Ref
	Urban	113	87	200		0.61 (0.41–0.92)	4.78 (1.81–12.65)
Education	≤Primary	177	87	264	6.89 (p = 0.009)	Ref	Ref
	≥Secondary	72	64	136		0.57 (0.37–0.87)	0.66 (0.31–1.42)
Belief as holistic treatment	No	80	76	156	17.73 (p = 0.001)	Ref	Ref
	Yes	169	75	244		2.14 (1.41–3.24)	1.19 (0.34–4.19)
High cost of CM	No	62	80	142	32.37 (p<0.001)	Ref	Ref
	Yes	187	71	258		3.40 (2.21–5.22)	6.80 (2.10, 22.04)
Accessibility of TM	No	65	96	161	54.88 (p<0.001)	Ref	Ref
	Yes	184	55	239		4.94 (3.20–7.64)	11.18 (4.03–31.00)
Effectiveness of TM	No	17	49	66	44.80 (p <0.001)	Ref	Ref
	Yes	232	102	334		6.56 (3.60–11.93)	3.45 (1.05–11.37)
Less side effects of TM	No	100	88	188	12.39 (p<0.001)	Ref	Ref
	Yes	149	63	212		2.08 (1.38–3.14)	2.58 (1.06–6.28)
Harmful effect of CM	No	143	84	227	0.12 (p = 0.725)	Ref	Ref
	Yes	106	67	173		0.93 (0.62–1.40)	0.01 (0.003–0.044)
Culture	No	158	112	270	4.92 (p = 0.027)	Ref	Ref
	Yes	91	39	130		1.65 (1.06–2.58)	0.60 (0.26–1.39)
Testimonials	No	22	65	87	64.64 (p <0.001)	Ref	Ref
	Yes	227	86	313		7.80 (4.53–13.43)	7.55 (2.75–20.69)
Low cost of TM	No	145	119	264	13.09 (p<0.001)	Ref	Ref
	Yes	104	32	136		2.67 (1.68–4.24)	10.48 (4.30–25.54)
Religious belief	No	162	65	227	18.56 (p<0.001)	Ref	Ref
	Yes	87	86	173		0.41 (0.27–0.61)	0.09 (0.04–0.21)
Lack of information	No	38	95	133	96.17 (p<0.001)	Ref	Ref
	Yes	211	56	267		9.42 (5.84–15.19)	13.82 (4.62–41.33)

## Discussion

The findings of this study provide valuable insights into the multiple factors influencing the use of traditional healing practices for jaundice in Bangladesh. Seeking care is acknowledged as a complex behavior shaped by various influences [24]. Our results also highlight the complex interplay of sociocultural, economic, and geographical determinants that shape treatment-seeking behaviors in both rural and urban areas.

The role of traditional healers in society extends beyond mere treatment provision. Our qualitative study reveals that these healers often act as trusted advisors and bearers of cultural traditions, which has led to their widespread acceptance, particularly in rural areas. A study in Ethiopia similarly observed that trust and cultural acceptance are key factors driving the preference for traditional medicine [25]. This social role influences people to seek out their treatments, even in places where modern medicine is available.

The prevalence of traditional healing practices for jaundice was found to be substantial, with 62% of the study population favoring TM. This high prevalence is consistent with the findings of a study that reported that 59% of jaundiced patients in Matlab, Bangladesh, seek traditional healing methods [20]. The present study found a diverse range of traditional healing methods used as a treatment for jaundice. These diverse findings align with other research showing various uses of TM practices in Bangladesh, Pakistan and India [13–15, 26–29].

The most common traditional treatments found in this study were scalp and hand cleansing rituals (45.78%), using herbal remedies (37%), and wearing jaundice garlands (23%). This pattern aligns with another study conducted in Bangladesh, which found that hand washing was practiced by 56% of participants, garlanding by 24%, and drinking herbal juices by 32% [20]. However, our study is the first to document the prevalence of several unique traditional treatments, including liver khilano (feeding the liver), liver katano (cutting the liver), cauterization, chanting over lemons on an earthen oven, ear candling, Pet tana (abdominal suction with a horn), and chanted mud showers. These novel findings contribute to understanding diverse traditional healing practices and reflect the holistic approach of traditional healing, addressing physical symptoms with spiritual and emotional aspects of health.

Traditional healing practices varied across different regions of Bangladesh, influenced by the availability of specific plants and contents. For instance, garland use was more common in villages compared to urban areas because the necessary plants, like Bamanhati (Turks Turban), Bhringraj (false daisy), and Apang (chaff-flower), grow in local jungles [30, 31]. This study’s findings on jaundice garlands are similar to those in another study in Bangladesh [32]. Plants and bark are also used in India as jaundice garlands [33]. In Nepal’s Morang district, people also use spiritual rituals and flower garlands to treat jaundice [34]. Another variation of the jaundice garland is made in Nepal using sun-dried rhizomes of *A*. *calamus* [35].

The study found that a significant portion of patients (37%) used herbal medicine for their treatment. Plants, barks, or leaves of 87 ethnomedicinal species belonging to 51 different families, including *Ehretia laevis*, *Amaranthus spinosus L*., *Cissampelos pareira L*., and *Acalypha*, are commonly consumed orally to treat jaundice [33, 36]. While some ethnopharmacological studies have highlighted the effectiveness of various medicinal plants and herbs, contemporary evidence-based medical research has raised concerns about potential adverse effects on the liver, such as herb-induced liver injury (HILI) and hepatotoxicity [37, 38]. Therefore, there is a need for careful monitoring and regulation of traditional treatments to prevent unintended harm, especially given the delicate nature of liver health.

The push-pull framework revealed several push factors driving patients away from CM, including high costs, perceived ineffectiveness for jaundice, and fear of side effects. Conversely, the pull factors attracting patients to TM were particularly strong and diverse. Key pull factors include the perceived effectiveness, safety, and affordability of traditional treatments. Belief in TM’s effectiveness for jaundice, often supported by personal testimonials, was a powerful motivator (OR: 7.55). Qualitative findings of this study also disclosed that individuals frequently sought TM when they were unsatisfied with conventional medicine, suggesting a significant level of community trust in these healers. This trend is consistent with other research showing many switch to TM after initial failures with conventional healthcare [15, 39]. However, our findings also revealed a reverse pattern. All participants initially using traditional methods ultimately sought conventional medical care at the study hospital. This suggests that while TM was the primary healthcare choice for many, conventional treatment was considered a last resort for them.

Lack of information about CM for jaundice was strongly associated with the use of TM (OR: 13.82), suggesting that information and communication greatly influence patient choices. Accessibility of traditional healers (OR: 11.18) and lower costs of TM (OR: 10.48) were additional significant factors that strongly influenced the use of TM. These findings are consistent with other studies in Bangladesh, where effectiveness, fewer adverse effects, affordability, and lower costs were the most common reasons for using CAM in Bangladesh [13, 14]. This highlights the need to improve access and affordability in the conventional medical system to encourage patients to choose CM. Although the government subsidizes most treatments and diagnostic tests in public hospitals in Bangladesh, providing free or low-cost care, many still perceive TM as more affordable than CM and prefer traditional treatments [14]. This suggests a general lack of awareness about the availability of government healthcare services, which leads people to conflate expensive private care with affordable public services. Cultural and religious beliefs also influenced treatment choices, though they had a less significant impact in the adjusted analysis. This suggests that while cultural factors play a role, practical considerations such as cost, accessibility, positive testimonials, and perceived effectiveness strongly influence decision-making [13, 14].

In this study, some sociodemographic factors played a significant role in treatment preferences as the personal factors. Older adults (71% for 50+ years), rural residents (68%), and individuals with no or primary education (72%) were more likely to go for traditional treatments. These findings are consistent with other studies that have found age, education, and rural residence associated with higher use of TM [39]. Another study reported that 75 to 80% of the population in rural and semi-urban areas favor traditional approaches for various health conditions [40]. The association between lower education levels and preference for TM suggests that health literacy plays an important role in treatment choices.

Contrary to common assumptions [15], the use of TM was not confined to lower socioeconomic groups or areas with limited access to conventional medicine found in this study. While our study found that rural residents (68% of 200 rural people) were more likely to use traditional methods, a considerable percentage (57% of 200) of urban people with access to modern healthcare also incorporated these practices. Unadjusted logistic regression analysis showed that urban residents were less likely to use TM, possibly due to better access to conventional healthcare. However, after adjusting for socioeconomic factors, perceptions, and healthcare accessibility, urban residents showed a significantly higher likelihood of using TM. This shift highlights the complexity of factors influencing healthcare choices beyond simple urban-rural divides. Our study found no significant association between income and treatment choice for jaundice. This can be explained by the fact that when presented with both options (TM and CM), many people choose traditional methods due to perceived lower costs coupled with better efficacy and fewer side effects. This perception of cost-effectiveness, rather than absolute affordability, is a driving factor in treatment decisions across different income levels. This aligns with Andersen’s Behavioral Model of Health Services Use, which suggests that access to healthcare is influenced by some predisposing factors, enabling resources, and need-based considerations [24, 41]. In our study, enabling factors like proximity and cost play a crucial role in shaping treatment preferences, particularly in rural Bangladesh, where traditional medicine is often viewed as more accessible and affordable.

Interestingly, our study found that 93% of patients who visited the traditional healer and 68% of those who used conventional medicine (accounting for 78% overall) believed that TM effectively cures jaundice. This belief persisted despite most of them experiencing no improvement in their jaundice symptoms. Observing others who reportedly recovered using traditional methods contributed to this widespread belief in TM. To understand why such beliefs are prevalent, it is important to consider the nature of common causes of jaundice in Bangladesh. Hepatitis A (HAV) and Hepatitis E (HEV) viruses spread through the fecal-oral route are associated with poor sanitation and cause viral hepatitis and symptoms of jaundice [42]. In Bangladesh, HEV is endemic and the leading cause of acute jaundice [2]. Another study found that HAV and HEV contribute to a total of 29% of cases of acute jaundice in Bangladesh [3]. However, acute HAV and HEV are usually self-limiting; patients typically recover from jaundice symptoms within 15 to 65 days without requiring treatment [42]. Focus group discussions revealed that healers typically tell patients their symptoms should improve within three weeks of their treatment, coinciding with the duration of natural resolution of most acute viral hepatitis cases. Therefore, when patients with self-limiting forms of jaundice visit traditional healers, any subsequent improvement is likely coincidental. The jaundice symptoms typically subside over time naturally, irrespective of the healer’s treatments. This misattribution, where natural recovery is credited to TM, reinforces the belief in its efficacy against jaundice.

### Implications

Our findings have significant implications for health policy and healthcare delivery systems in Bangladesh and similar countries where TM is commonly used to treat jaundice. A key implication is the need for targeted health education and awareness programs focusing on jaundice, particularly aimed at individuals aged 50 or more, rural populations, and those with no or low education levels since they are more likely to rely on TM. The objective of these programmes should be to dispel misconceptions, clarify the causes, symptoms, and potential complications of jaundice, and explain the benefits and procedures of both TM and CM to support informed treatment choices.

Another significant implication is the need to improve access to contemporary healthcare services, especially in government healthcare facilities in rural areas. Telemedicine services, which are already in operation in Bangladesh, along with mobile health clinics, could be expanded to reach remote populations. Additionally, community health clinics should equip health workers with the appropriate training and scientific knowledge to bridge the gap between traditional and conventional medicine.

Cultural sensitivity in healthcare is another crucial implication of this study. Healthcare providers should be trained in cultural competency to understand and respect patients’ beliefs, preferences, and values and make jaundice treatment more holistic and culturally acceptable. Involving community members and traditional healers in jaundice awareness programs can enhance effectiveness. Fostering communication between conventional doctors and traditional healers may also improve mutual understanding and cooperation, leading to a more cohesive treatment approach. By considering these points, healthcare systems in Bangladesh and similar countries can adopt a unified, culturally sensitive, and effective strategy for treating jaundice, ultimately improving health outcomes.

### Limitations

The participants for the quantitative survey were recruited from a specific hospital, which might introduce selection bias into the sample. This selection procedure may not accurately reflect all jaundice patients nationwide, especially those not seeking hospital treatment. Such a sample may skew the study results towards people with access to hospital care, affecting the generalizability of the findings. Self-reported data collection could lead to recall bias and social desirability bias. Participants might have reported behaviors or opinions they deemed socially acceptable or expected rather than their actual behaviors or beliefs.

## Conclusions

The study concludes that both push and pull factors significantly influence jaundiced patients in Bangladesh to prefer TM over CM. The choice of treatment modality is not solely based on medical factors but is deeply influenced by personal, social, and geographical contexts. Barriers to CM, including high costs and fear of side effects, serve as significant push factors driving patients toward TM. Key pull factors attracting individuals to TM include trust in healers, affordability, accessibility, personal testimonials, cultural beliefs, and perceived effectiveness and safety, especially among individuals aged 50 or more, rural residents, and those with lower education levels. However, the patients eventually turn to conventional healthcare when symptoms persist.

## Supporting information

S1 Dataset (CSV)
